# Social Wellbeing in Cancer Survivorship: A Cross-Sectional Analysis of Self-Reported Relationship Closeness and Ambivalence from a Community Sample

**DOI:** 10.3390/curroncol30020133

**Published:** 2023-01-31

**Authors:** Chiara Acquati, Ellen Miller-Sonet, Anao Zhang, Elena Ionescu

**Affiliations:** 1 Graduate College of Social Work, University of Houston, Houston, TX 77204, USA; 2 Department of Clinical Sciences, Tilman J. Fertitta Family College of Medicine, University of Houston, Houston, TX 77204, USA; 3 Department of Health Disparities Research, The University of Texas MD Anderson Cancer Center, Houston, TX 77030, USA; 4 Cancer*Care*, New York, NY 10001, USA; 5 School of Social Work, University of Michigan, Ann Arbor, MI 48109, USA

**Keywords:** quality of life, close relationships, social wellbeing, cancer survivorship, psychosocial oncology, survivorship care, patient-reported outcomes

## Abstract

Improvements in early screening and treatment have contributed to the growth of the number of cancer survivors. Understanding and mitigating the adverse psychosocial, functional, and economic outcomes they experience is critical. Social wellbeing refers to the quality of the relationship with partners/spouses, children, or significant others. Close relationships contribute to quality of life and self-management; however, limited literature exists about social wellbeing during survivorship. This study examined positive and negative self-reported changes in a community sample of 505 cancer survivors. Fourteen items assessed changes in communication, closeness with partner/children, stability of the relationship, and caregiving burden. An exploratory factor analysis was conducted using a robust weighted least square procedure. Differences by sociodemographic and clinical characteristics were investigated. Respondents were mostly male, non-Hispanic white, and ≥4 years since diagnosis. Two factors, labeled Relationship Closeness and Ambivalence, emerged from the analysis. Women, younger survivors, individuals from minority groups, and those with lower income experienced greater negative changes in social wellbeing. Variations by treatment status, time since diagnosis, and institution were also reported. This contribution identifies groups of cancer survivors experiencing affected social wellbeing. Results emphasize the need to develop interventions sustaining the quality of interpersonal relationships to promote long-term outcomes.

## 1. Introduction

The implementation of early cancer screening and detection, combined with advances in curative treatment options, have contributed to the continued growth of the number of cancer survivors living in the United States [1,2]. To date, estimates from the National Cancer Institute and the American Cancer Society indicate that there are 16.9 million cancer survivors in the country, accounting for 5% of the population [3]. Additionally, the number of cancer survivors is expected to increase to 26.1 million by 2040 [4]. Physical, emotional, and financial consequences are experienced well into survivorship [5,6,7,8,9,10]. About 60% of survivors report persistent distress and fear of recurrence [5,8], approximately 36.5% remain unable to work [11,12], and between 15% and 75% present cancer-related cognitive impairment [2,13,14]. As a result, it becomes imperative to better understand the experience of this heterogeneous group and to identify strategies and approaches to address their unmet needs and long-term issues, whether due to treatment side effects, disparities, or social determinants of health [7,15,16,17,18]. To this end, cancer survivorship research has emerged as a subset of efforts aimed at understanding the psychosocial sequelae associated with cancer treatment and to prevent and mitigate multifaceted adverse outcomes [9,19,20,21,22].

An extensive body of evidence has demonstrated the pervasive consequences of the illness for close relationships, in terms of mental health, communication, and relationship dissolution [7,23,24,25,26]. Elevated rates of psychological distress impair the quality of life of survivors and their partners [27,28,29]. Relationship satisfaction was reported among couples engaging in mutual constructive communication, expression of feelings, and negotiation [30]. On the contrary, avoidance, holding back, or disengagement have been linked to poorer relationship functioning, coping, and psychological wellbeing [30] Cancer-related distress and caregiving responsibilities may also negatively alter relationship stability, with greater odds of separation/divorce recorded among female survivors, young adults, and those experiencing greater distress and financial problems [31,32,33]. Although a recent systematic review documented that cancer is linked to a small decrease in divorce rate [34], Nalbant et al. (2021) found that cancer was the main reported cause for relationship dissolution among partners of cancer survivors [35].

While quality of life is a broad multidimensional concept—often resulting from the “individual’s perception of their position in life in the context of the culture and value system in which they live and in relation to their goals, expectations, standards, and concerns” [36], social wellbeing refers to the satisfaction the individual has in regard to the quality of the relationships with others [37,38,39]. Authors that investigated patient-reported outcomes in cancer survivorship found that close relationships are contributing factors for quality of life after diagnosis. Support from partners, family members, and the larger social network protects against physical morbidity and mortality, while also promoting psychological wellbeing and self-management [7,37,40,41,42,43,44]. Yet, adverse physical and psychosocial consequences of cancer have the potential to impair survivors’ social wellbeing [7,23,24,45]. Contributions have documented that cancer survivors tend to show decreased social functioning because of late treatment side-effects, impaired physical functioning, mental health symptomatology, perceived stigma, financial hardship, and access to and changes in their social networks [31,46,47,48].

Social wellbeing in cancer survivorship varies by gender, age, ethnic and cultural aspects, type/stage of cancer, and socioeconomic and personality characteristics [9,26,49,50,51]. Although most survivors are older than 65 [1], a substantial number of patients are diagnosed with cancer during young adulthood or adulthood, with differential effects on their psychosocial outcomes [6,41,52]. Studies have shown that older age is both an aggravating and a protective factor [40,41,52]. While older patients experience more comorbidities and social isolation, they appear to cope better with the impact of the disease on close relationships as they are more inclined to preserve or improve existing ones [40,52,53]. Younger survivors, on the contrary, are faced with the premature confrontation with mortality, disruption of educational and professional goals, financial difficulties, and reproductive and sexual health concerns, which lead to difficulties in maintaining or establishing romantic partnerships and intimacy [23,25]. While there is still scarce literature concentrating on cancer survivors from minoritized racial/ethnic groups and their social wellbeing, studies have shown that worse outcomes were reported, especially for Hispanic patients [54], and that culturally informed and contextual factors guide family interactions and coping [4,55].

Despite growing attention to social wellbeing after cancer and the development of intervention approaches that capitalize on the relationship with significant others to alleviate the burden of the illness [21,53,56], gaps remain in our understanding of patterns and quality of close relationships beyond active treatment, next to the inclusion of community-based samples able to illustrate the experience of survivors from different backgrounds, race/ethnicities, and receiving care in diverse oncology settings. The present study aims to examine positive and negative self-reported changes in social wellbeing by sociodemographic and clinical characteristics.

## 2. Materials and Methods

### 2.1. Procedure

This contribution is a secondary data analysis of the Survivorship Survey data collected between July and December 2015 from Cancer*Care*, a leading US nonprofit organization providing professional supportive services including counseling, support groups, educational workshops, and financial assistance to cancer survivors and caregivers. Survivors were recruited through online panels; respondents were limited to individuals who were 25 years of age and older, and who had received a confirmed diagnosis of cancer from a physician/healthcare professional. Fifty percent of the sample included common cancers (lung, breast, colorectal, and prostate), and research vendors utilized specific criteria and filters so that approximately 25% of respondents were recruited from each region of the nation (Northeast, Midwest, Southeast, and Southwest/West) to increase sample representativeness. Approximately 3000 participants were invited by e-mail to reach the target sample of 500 respondents, and 505 answers were collected for the survivorship questionnaire. To minimize response biases, potential participants were not selected from cancer survivors who have used the services of the organization, online communities, or client database. Informed consent was obtained from all individual participants included in the original study. All procedures were in accordance with the ethical standards of the institutional research committee and with the 1964 Helsinki declaration and its later amendments. The dataset inclusive of variables and measures of interest for the present work was shared with the research team after IRB approval (19 June 2018).

### 2.2. Measures

#### 2.2.1. Impact of Cancer on Relationships

A total of 14 items were utilized to assess differences in the social wellbeing of the participants since the cancer diagnosis. Items were initially developed by social work counselors and subsequently reviewed by an advisory board inclusive of experts in survey development and patient care. Using dichotomous answer options (yes/no), cancer survivors were asked to ascertain whether the illness contributed to positive or negative changes in different aspects of their lives: communication (more meaningful conversation with loved ones, more likely/less likely to share their thoughts and feelings with loved ones), closeness with partner and children (time spent with partner, time spent with children, level of intimacy, sense of isolation, children becoming too attached or withdrawn/angry), stability of the relationship (divorce or relationship dissolution with spouse/partner), and caregiver burden (partner/spouse being exhausted because of extra responsibilities, having trouble being dependent on others).

#### 2.2.2. Demographic and Clinical Information

Demographic characteristics such as age, sex, ethnicity/race, education, annual household income, and healthcare insurance were self-reported. Clinical factors assessed as part of the survey included cancer type, time since diagnosis, current cancer status, treatment type, and information about the institution where participants received care.

### 2.3. Data Analysis

Descriptive statistics were obtained to summarize the sample’s characteristics. Means and standard deviations were calculated for continuous variables, while frequency and percentages have been used for categorical variables. An exploratory factor analysis (EFA) was conducted on the 14 items that asked participants to rate positive and negative changes in their relationships with partners/spouses, family members, and children. Then, the resulting pseudo-factors obtained by summing items loading on the two-factor solution were compared by socio-demographic and clinical characteristics using chi-square tests for nominal variables and ANOVAs for continuous variables. We also calculated post hoc Tukey’s test for comparison between individual groups as well as Cohen’s *d* effect size when appropriate. Bonferroni correction was conducted for all analyses. *Mplus* version 7.31 was utilized for data cleaning, management, and analysis [57]. The level of significance was set at *p* < 0.05.

## 3. Results

### 3.1. Sample

The characteristics of the sample are presented in Table 1. A total of 505 participants were included. Half of the respondents identified as male (52.9%) and non-Hispanic white (65.9%), and they were young adult cancer survivors below the age of 44 years (39.2%). Most of the participants were college graduates (60.8%), declared an annual income of over 50,000 USD (68.3%), and had health insurance (97.2%). The most reported cancer types were prostate (13.7%), early-stage breast (13.1%), colorectal (8.9%), and gynecological (7.3%). Participants had received multiple forms of treatment (56.2%), and they were not undergoing maintenance therapy when the survey was completed (40.4%). Respondents were diagnosed more than 4 years earlier (long-term survivorship 34.9%), with one-fourth of the sample been diagnosed within the previous 2 years (short-term survivorship, 25.0%). Most cancer survivors received care at academic cancer centers (29.7%) and in community hospitals (30.8%).

Examination of the demographic variables by age category revealed that the Black/African American and Hispanic categories tended to be mostly present in the younger age group, with older participants being mostly non-Hispanic white (χ^2^_(6)_ = 118.98; *p* < 0.0001). Males were under-represented in the middle-age group, while females were over-represented among adults (χ^2^_(2)_ = 17.49; *p* < 0.01). Significant differences were identified by treatment status (χ^2^_(4)_ = 18.68; *p* = 0.0009), with multiple treatments more frequently reported by younger patients (χ^2^_(1)_ = 14.39; *p* = 0.00015). Additionally, insurance status varied by age group (χ^2^_(2)_ = 7.65; *p* = 0.02), and younger survivors were more likely to be lacking coverage. No significant differences were detected for income (*p* = 0.7) and education (*p* = 0.06).

### 3.2. Exploratory Factor Analysis

As an initial step, an exploratory factor analysis (EFA) was performed on the 14 items assessing self-reported changes in close relationships. As the items were dichotomous, a robust weighted least square procedure was used, and the initial factor solution was rotated using the GEOMIN oblique method [57]. Up to four factors were extracted, according to the previously hypothesized domains (communication, closeness with partner/children/family members, stability of the relationship, and caregiver burden). An overview of the items, different models, and the two-factor structure loadings is available in Supplementary Materials Tables S1 and S2. Model 1, using a single factor, resulted in a poor fit (χ^2^_(77)_ = 469.22; *p* < 0.0001). Four items (item 4, 11, 12, and 14) were not significantly related to the single factor. Model 2 utilized a two-factor solution and was a significant improvement over the one factor model (χ^2^_(13)_ = 213.53; *p* < 0.0001). Items 2, 4, 5, 7, 12, and 14 had significant loadings on both factors; however, in all cases, one loading was negative and/or substantially smaller than the other. The three-factor solution (Model 3) showed only a minimal improvement (χ^2^_(52)_ = 120.47; *p* < 0.0001), and all but four of the items (items 7, 11, 13, and 14) had significant cross loadings. Lastly, a four-factor solution (Model 4) was only minimally better than the three-factor option (χ^2^_(41)_ = 89.78; *p* < 0.0001) with six items (items 4, 5, 6, 7, 8, and 10) indicating significant cross loadings and similar loadings on two factors. Despite the best model fit, the four-factor solution did not match the previously hypothesized domains. Because of the sensitivity of the chi-square test to the large sample size, it was decided to utilize a two-factor solution considering the best conceptual model and the empirical model fit. To provide a simple description that could easily be replicated by other studies, the two factors were created by summing the items with the highest loadings on each factor (≥0.45), which were then labeled Relationship Closeness and Relationship Ambivalence. The correlation between the two factors was examined (r = 0.23, *p* < 0.05), and internal consistency was investigated (Cronbach’s alpha for relationship closeness, α = 0.65; Cronbach’s alpha for relationship ambivalence, α= 0.57). Mean scores (ranging from 0 to 2) were then compared by variables of interest, with results presented below.

### 3.3. Differences in Relationship Closeness and Ambivalence by Sociodemographic Characteristics

**Figure 1:**
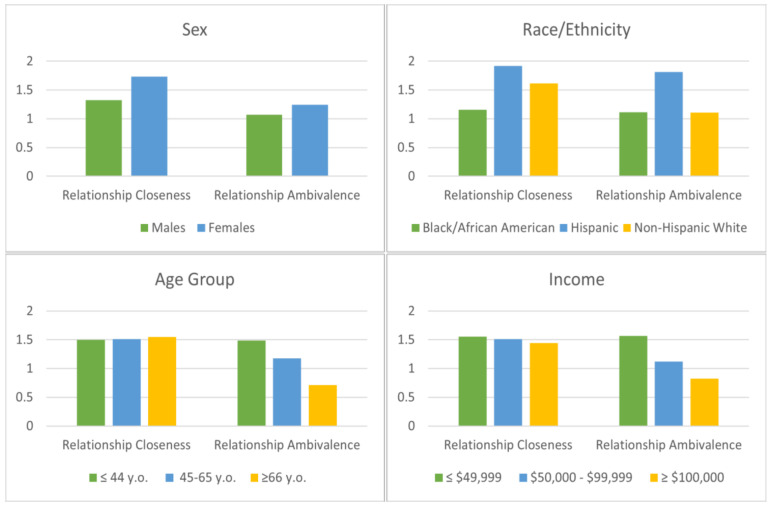
Mean scores for relationship closeness and ambivalence by key sociodemographic variables.

Figure 1 illustrates mean scores for relationship closeness and ambivalence compared by sociodemographic variables. Significant differences were identified between male and female respondents for both closeness (F _(1, 503)_ = 10.04; *p* = 0.0016; R^2^ = 0.02) and ambivalence (F _(1, 503)_ = 4.04; *p* = 0.0451, R^2^ = 0.008), with female survivors reporting higher levels of positive (Cohen’s d = 0.28) and negative changes in social wellbeing (Cohen’s d = 0.18) than males. Race was also significantly correlated with both positive (F _(4, 500)_ = 3.32; *p* = 0.0106; R^2^ = 0.026) and negative effects (F _(4, 500)_ = 3.54; *p* = 0.007; R^2^ = 0.027). Hispanic cancer survivors and non-Hispanic whites reported significantly greater closeness than Black/African American participants (Cohen’s d = 0.53, Cohen’s d = 0.32, respectively). Relationship ambivalence was more elevated among survivors who identified as Hispanic than Black/African American (Cohen’s d = 0.59) and non-Hispanic white respondents (Cohen’s d = 0.58).

Although there were no differences in positive changes by age group (F _(2, 502)_ = 0.04 *p* = 0.9597, R^2^ = 0.0001), variations existed in terms of relationship ambivalence in the illness aftermath (F _(2, 502)_ = 11.01; *p* < 0.0001, R^2^ = 0.042), suggesting greater vulnerability for survivors diagnosed at a younger age. The oldest age group (≥66 years) had fewer negative changes than either young adult survivors (Cohen’s d = 0.53) or the middle-age group (Cohen’s d = 0.33), but these two groups did not differ significantly (Cohen’s d = 0.20).

Lastly, while relationship closeness did not differ by income (F _(2, 471)_ =0.20; *p* = 0.821, R^2^ = 0.001), cancer survivors from low socioeconomic backgrounds were significantly more likely to report negative consequences (F _(2, 471)_ = 8.65; *p* = 0.0002; R^2^ = 0.035). Those with the lowest income (≤49,999 USD) had more elevated ambivalence than the middle (50,000–99,999 USD; Cohen’s d = 0.26) and higher income (≥100,000 USD; Cohen’s d = 0.51) groups. No significant differences were registered for education level, insurance coverage, and geographical locations.

### 3.4. Differences in Relationship Closeness and Ambivalence by Clinical Characteristics

**Figure 2:**
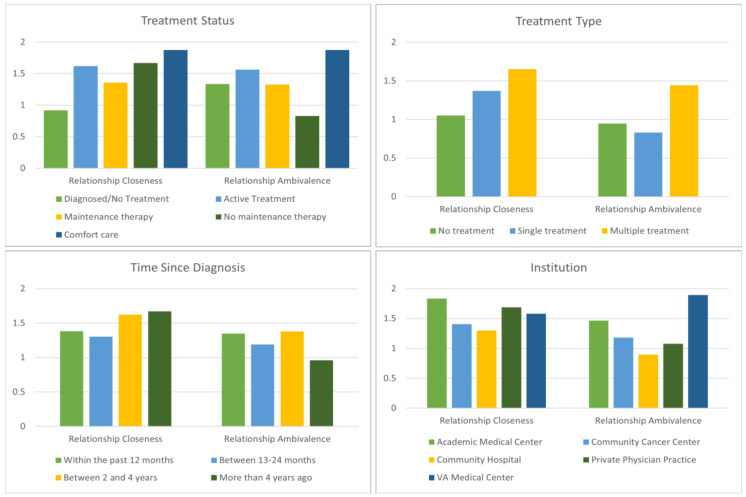
Mean scores for relationship closeness and ambivalence by key clinical characteristics.

Figure 2 illustrates mean scores for relationship closeness and ambivalence by clinical variables. No differences were detected by cancer type; a result that may be due to the large number of cancers included. When cancer status was examined, significant differences for positive changes in social wellbeing (F _(5, 499)_ = 2.25; *p* = 0.0481, R^2^ = 0.018) were identified. Post hoc analysis revealed that individuals who had completed treatment and were not on maintenance therapy presented greater closeness than those who were diagnosed but not yet receiving treatment (mean difference = 0.75, *p* < 0.05). Similar results emerged when ambivalence was investigated; significant variations existed between those on active treatment and cancer survivors who completed treatment, as well as between survivors on maintenance therapy and those who were not (F _(5, 499)_ = 5.312; *p* < 0.001, R^2^ = 0.023).

Significant differences for positive (F _(2, 502)_ = 3.25; *p* = 0.0394, R^2^ = 0.013) and negative changes existed by treatment category (F _(2, 502)_ = 13.53; *p* < 0.0001; R^2^ = 0.051); respondents who received multiple treatments tended to report greater closeness than those who had received a single treatment approach (Cohen’s d = 0.19), yet the multiple treatment group also had significantly higher rates of ambivalence (Cohen’s d = 0.47). More negative effects were found according to time since diagnosis (F _(3, 501)_ = 2.682; *p* = 0.046, R^2^ = 0.009), with a trend toward significance for the difference between short (2–4 years) and long-term survivorship (mean difference = 0.417, *p* = 0.061). Lastly, variations were assessed by institution (F _(5, 485)_ = 3.686; *p* = 0.003, R^2^ = 0.075); fewer negative outcomes were identified for those treated at community hospitals when compared to those treated at academic medical centers (mean difference = 0.571, *p* = 0.005) and Veteran’s Administration agencies (mean difference = 1.001, *p* = 0.035).

## 4. Discussion

The present work extends current literature on social wellbeing in cancer survivorship, by investigating self-reported positive and negative changes in the context of close relationships. Two factors, Relationship Closeness and Relationship Ambivalence were identified via exploratory factor analysis. Then, differences by sociodemographic and clinical characteristics were examined. Results indicate that women, younger survivors, Black and Hispanic survivors, and those with lower income presented more impaired social wellbeing. Additionally, variations were registered by treatment status, time since diagnosis, and institution.

The study confirms existing literature investigating social outcomes in cancer survivors. Female participants reported both greater negative and positive changes in social wellbeing. This finding can be linked to reported sex and gender differences in morbidity and adjustment [10], as well as to emerging application of theoretical frameworks that contribute to describe gender-related differences [58]. For instance, Social Role Theory can characterize this finding as resulting from perceived role and caregiving responsibilities [58], while transactional approaches may relate this to differential appraisals [59]. Although no differences for closeness were detected by age group, greater ambivalence among younger survivors confirms the profound psychosocial impact of facing cancer as a young adult. Studies have consistently documented the clinical decrement of social functioning over time, especially for young survivors experiencing greater symptomatology, limited social support [48,60,61], and higher distress in their relationships [23,48,62,63,64,65]. Three recent systematic reviews identified that this group continues to experience difficulties establishing and maintaining relationships with peers, family members, and partners [64,65,66].

In addition to sex and age, members of minoritized groups and socioeconomically vulnerable individuals experienced higher levels of ambivalence. These findings can contribute to illustrate the differential impact of the illness for those who experience cancer from a position of vulnerability. Financial hardship [67,68,69,70,71,72] can affect psychological distress, quality of life, and social relations [73]. Worse outcomes, in the form of lower closeness and higher ambivalence, were reported by Black/African American and Hispanic respondents, respectively. These results reflect the intersection of social determinants of health [17] and culturally informed expectations for family interaction and provision of support [54,55], which require greater understanding by the literature and multilevel interventions [16,56].

The transition to survivorship is confirmed to be a delicate moment for the social wellbeing of the individual, as evidenced by significant variations in relationship ambivalence between those on active treatment and cancer survivors who completed treatment, as well as between survivors on maintenance therapy and those who were not. Previous evidence has revealed cancer survivors and their partners’ tendency to withdraw from each other in the period immediately following the end of active treatment [24]. Differences in negative consequences by treatment modality, status, and time since diagnosis can help in identifying moments of potential susceptibility for the social wellbeing of survivors; in this sample, active treatment, maintenance therapy, and the early survivorship phase were characterized by greater ambivalence. This result can assist future efforts to identify and intervene on psychosocial resources that contribute to the wellbeing of both patients and caregivers [21,22,56]. As greater survival rates have been reported for those treated at NCI-designated comprehensive cancer centers [74,75], it was unexpected to register fewer negative outcomes for those that received care at community hospitals. While this finding may be due to this sample’s characteristics, Zebrack et al. [76] found that providers at community cancer programs presented greater institutional capacity for continuity in the delivery of psychosocial care over time. Future contributions investigating the implementation and outcome evaluation of comprehensive psychosocial support services across cancer care settings are needed.

The cross-sectional design, the utilization of self-reported dichotomous items, and the lack of a comparison group of healthy peers are important limitations that affect the present work. Positive and negative variations in social wellbeing were evaluated using a list of dichotomous items created for the purpose of the survey by providers. Hence, it was not possible to discriminate among the different domains affected by the illness, nor to elaborate on the amount of change participants experienced since diagnosis. The inclusion of standardized and validated questionnaires is, therefore, recommended for future studies. Furthermore, SEM model fit indices were acceptable but lower than ideal, suggesting future research to consider alternative models when additional measures are available to describe social wellbeing in the cancer aftermath. The lack of a comparison group of healthy peers prevented the authors from inferring whether these changes occurred due to illness, aging process, or other sample characteristics. Furthermore, the survey was cross-sectional, and it was not possible to elaborate on causation nor on trajectories of positive and negative changes over time and at critical turning points of the cancer continuum. Similarly, the association with mental health data should be further investigated to clarify whether variations in social wellbeing were linked to affected mood or distress. While the inclusion of a large, national, and diverse sample is an aspect of strength of the present analysis, recruitment via online panels led to the overrepresentation and underrepresentation of certain groups of survivors, which may have influenced some of the current results.

## 5. Conclusions

The present study revealed that there are groups of cancer survivors experiencing more affected social wellbeing: women, young adults, individuals from minoritized groups, and those with lower financial resources. Furthermore, variations by treatment status, time since diagnosis, and institution suggest that social wellbeing may be influenced by the interaction with the healthcare system. Specifically, our findings indicate that there may be settings not fully equipped to provide models of care encompassing the psychosocial needs of patients, which can ultimately affect their social relationships. This work also has implications for oncology social workers and healthcare teams involved in direct care delivery. Results emphasize the need to enhance providers’ capacity for addressing psychosocial issues related to the relationship with partners, family members, and the larger social network. At the same time, this contribution unveiled the necessity to develop interventions able to sustain the quality of survivors’ interpersonal relationships and overall social wellbeing, with a particular emphasis on the experience of certain groups and for the differential burden that accompanies active treatment, early vs. long-term survivorship. Future research should expand both qualitatively and quantitatively current understanding of the experience of survivors reporting more affected social wellbeing and investigate the development and implementation of supportive care services alleviating stressors impairing the quality of close relationships.

## Figures and Tables

**Table 1:** Sociodemographic and clinical characteristics of the sample (N = 505).

Variable	N	%
Sex		
Female	238	47.1%
Male	267	52.9%
Age (recoded in 3 groups)		
≤44	198	39.2%
45–65	179	35.4%
≥66	128	25.3%
Race/Ethnicity		
Asian	10	2.0%
Black/African American	115	22.8%
Hispanic	38	7.5%
Non-Hispanic white	333	65.9%
Other	9	1.8%
Income (USD)		
≤49,999	150	31.6%
50,000–99,999	204	43.0%
≥100,000	120	25.3%
Education		
Less than high school	6	1.2%
High-school graduate	63	12.5%
Some college	129	25.5%
Associate degree	62	12.3%
Bachelor’s degree	147	29.1%
Master’s degree	78	15.4%
Doctorate degree	20	4.0%
Insurance		
Insurance coverage	491	97.2%
Lack of insurance coverage	14	2.8%
Region in the US		
Midwest	116	23%
Northeast	129	25.5%
Southeast	133	26.3%
Southwest/West	127	25.1%
Cancer Type		
Prostate cancer	69	13.7%
Breast cancer (early stage)	66	13.1%
Colorectal cancer	41	8.1%
Endometrial, cervical, or ovarian cancer	37	7.3%
Thyroid	26	5.1%
Breast cancer (metastatic)	24	4.8%
Bladder	22	4.4%
Head and neck	21	4.2%
Kidney cancer	21	4.2%
Lymphoma	19	3.8%
Leukemia	18	3.6%
Brain tumor	17	3.4%
Liver cancer	16	3.2%
Melanoma	16	3.2%
Pancreatic	9	1.8%
Myeloma	8	1.6%
Stomach	5	1.0%
Other	45	8.9%
Treatment		
No treatment	19	3.8%
Single treatment	202	40.0%
Multiple treatment	284	56.2%
Time Since Diagnosis		
≤12 months	89	17.6%
13–24 months	126	25.0%
>2–4 years	114	22.6%
>4 years	176	34.9%
Treatment Facility		
Academic medical center	146	29.7%
Community cancer center	84	17.1%
Community hospital	151	30.8%
Private physician practice	77	15.7%
US Department of Veterans Affairs medical lefts	19	3.9%
Unsure	14	2.9%
Current Cancer Status		
Diagnosed but not yet treatment	36	7.1%
Active treatment	110	21.8%
Completed treatment and maintenance therapy	137	27.1%
Completed treatment and not on maintenance therapy	204	40.4%
Comfort care	8	1.6%
Other	10	2.0%

Note: Not all groups of n values and % add up to the reported sample size because of missing data.

## Data Availability

Primary data for this secondary analysis article were collected by Cancer*Care* as part of the Patient Access and Engagement Report. The datasets analyzed during the current study are available from the corresponding author on reasonable request.

## Supplementary Materials

The following supporting information can be downloaded at https://www.mdpi.com/article/10.3390/curroncol30020133/s1, Table S1. Exploratory factor analysis (EFA): comparison of fit indices; Table S2. Exploratory factor analysis (EFA): factor loadings.
