# Supplementary material for: Social Wellbeing in Cancer Survivorship: A Cross-Sectional Analysis of Self-Reported Relationship Closeness and Ambivalence from a Community Sample
Source: Curr Oncol. 2023 Jan 31;30(2):1720–32. doi: 10.3390/curroncol30020133 (PMC9955865; doi:10.3390/curroncol30020133)
Supplement: Supplementary file 1 [file curroncol-30-00133-s001.zip › curroncol-2155501-supplementary.pdf]

Supplementary Table S1. Exploratory Factor Analysis (EFA): comparison of fit indexes

	**χ^2**	**df (p)**	**CFI**	**RMSEA**	**TLI**	**SRMR**
Model 1 – Single factor	469.22	77(<.001)	.45	.10	.34	.19
Model 2 – Two factors	152.09	64(<.001)	.87	.05	.84	.10
Model 3 – Three factor	120.47	52(<.001)	.90	.05	.83	.09
Model 4 – Four factors	89.78	41(<.001)	.93	.05	.84	.08

Note. CFI = Comparative Fit Index; RMSEA = Root Mean Square Error of Approximation; TLI = Tucker–Lewis index; SRMR = Standardized Root Mean Square Residual

Supplementary Table S2. Exploratory Factor Analysis (EFA): factor loadings

**Items**	**Factor 1: Relationship Closeness**	**Factors 2: Relationship Ambivalence**
Item 1_More meaningful conversations with loved ones	**0.782**	0.002
Item 2_Spend more time with partner	**0.565**	-0.190
Item 3_More likely to share my thoughts and feelings with my loved ones	**0.815**	0.057
Item 4_Less likely to share my thoughts and feelings with my loved ones	-0.206	**-0.704**
Item 5_Decreased intimacy with my partner	0.061	**-0.533**
Item 6_Increased intimacy with partner	**0.479**	0.045
Item 7_Spent more time with my children or grandchildren	**0.696**	-0.349
Item 8_Have trouble being dependent on others	0.257	**-0.487**
Item 9_My partner resents the extra responsibilities they have because of my cancer	0.273	**-0.649**
Item 10_My partner is exhausted from the extra responsibilities they have because of my cancer	0.416	**-0.784**
Item 11_My partner and I have divorced or split up	0.023	**-0.299**
Item 12_I feel isolated from my family and loved ones	-0.162	**-0.599**
Item 13_My children have become too attached	0.441	**-0.509**
Item 14_My children have become angry and withdrawn	0.005	**-0.451**
